# The impact of sample retention and further analysis on doping behavior and detection: evidence from agent-based simulations

**DOI:** 10.3389/fspor.2025.1578929

**Published:** 2025-06-18

**Authors:** Daniel Westmattelmann, Marius Sprenger, Julian Lanfer, Benedikt Stoffers, Andrea Petróczi

**Affiliations:** ^1^ Center for Management, University of Münster, Münster, Germany; ^2^ School of Life Sciences, Pharmacy and Chemistry, Kingston University, London, United Kingdom; ^3^ Faculty of Health and Sport Sciences, Széchenyi István University, Győr, Hungary; ^4^ Willibald Gebhardt Institute, Universität Münster, Münster, Germany

**Keywords:** anti-doping, retesting, long-term storage, doping prevalence, doping behavior, agent-based modelling, deterrence theory, sample retention

## Abstract

**Introduction:**

Despite extensive testing efforts in anti-doping work, a persistent gap remains between low doping detection rates and substantially higher estimated doping prevalence in sports. Sample Retention and Further Analysis (SFA), which allows samples to be stored for up to ten years for future testing, offers a potential strategy to close this gap by increasing both detection and deterrence of doping.

**Methods:**

This study employs an agent-based modeling approach to simulate interactions among key stakeholders: athletes, anti-doping organizations, laboratories, and event organizers. The model captures athlete decision-making regarding doping, influenced by perceived sanction certainty and swiftness. SFA parameters, such as number of stored samples and duration of storage, were systematically varied to assess their impact.

**Results:**

Simulations show that increasing both the quantity of stored/retested samples and the storage duration reduces doping prevalence. A combined approach yields the strongest effect, with higher detection rates and lower doping behavior. However, regression analysis reveals diminishing returns at higher implementation levels, suggesting a non-linear effect.

**Discussion:**

The findings provide quantitative evidence that SFA enhances not only detection capacity but also deterrence by increasing the perceived long-term risk of sanctions. Effective SFA implementation requires strategic calibration to optimize impact. These results underscore the potential of SFA as a key component in anti-doping strategies and call for empirical validation and integration of additional behavioral factors in future research.

## Introduction

Deterrence against the use of prohibited substances in sports relies heavily on anti-doping testing and sanctions, such as bans from competition when doping is detected (1). Detection-based deterrence is a central paradigm of the global anti-doping system (2). This is demonstrated by approximately USD 500 million allocated to anti-doping efforts annually, with 48% of an Anti-Doping Organization's (ADO) budget spent on testing (3). However, the testing regimes of ADOs face criticism for their limited detection rates (4, 5). This is because the percentage of positive test results is rather low at around 0.7%–1.2% WADA (6–15), while estimates on doping prevalence in sports are considerably higher, with most ranging between 5% and 18% [for a systematic review on doping prevalence rates, see (16)]. Ideally, all instances of doping contributing to prevalence would be detected, aligning the prevalence rate with the incidence rate. However, current statistics indicate a considerable gap between these rates, highlighting the challenges faced by anti-doping efforts.

Thus, there is an ongoing debate about whether the effectiveness of anti-doping can be adequately evaluated based on detection rates (17). The effectiveness of anti-doping testing hinges on its ability to accurately capture true positives, which comprises two critical components: identifying all actual cases of doping (maximizing sensitivity) and ensuring that as few false positives occur as possible (maximizing specificity). However, the limited alignment between incidence rates (the percentage of positive test results) and estimated prevalence rates raises questions about the information value of testing statistics. Testing figures alone, such as the incidence rate, are not sufficient to comprehensively evaluate the effectiveness of anti-doping measures because testing serves a dual purpose: it not only detects doping cases but also acts as a deterrent against doping behaviors (17). Therefore, the success of anti-doping testing cannot be judged solely by the proportion of positive results it yields, as an effective deterrence mechanism reduces the prevalence of doping by discouraging athletes from engaging in such practices, irrespective of detection rates. Low incidence rates might not necessarily indicate ineffective testing; they could also reflect successful deterrence, where athletes are discouraged from doping due to the perceived risk of detection and associated penalties (18). Conversely, high incidence rates might indicate robust detection capabilities but could also suggest limited deterrence, as more athletes are willing to risk using prohibited substances. To accurately evaluate the effectiveness of testing, it is essential to consider both its ability to detect true positives and its broader impact on reducing the overall prevalence of doping in sport. Detection-based deterrence of testing can be perceived as a credible deterrent if the certainty of a test result turning out positive is perceived to be reasonably high (18, 19). This dual role emphasizes the importance of integrating detection capabilities with preventive strategies, such as education, to create a comprehensive and effective anti-doping framework. Therefore, it is unsurprising that a Delphi study with an international panel of experts from both academia and anti-doping practice identified the “effectiveness of anti-doping interventions” as the most critical focus area in the research agenda for doping prevention [(20), p. 7; (21)].

From the viewpoint of reliable doping detection, various conditions must be met to effectively detect doping. WADA guidelines addressed towards ADOs on how to implement effective testing regimes (22), supplemented by technical documents and letters [e.g., (23–25)] to address specific issues (e.g., sport specific analysis, Athlete Biological Passport, dried blood spot, specific drugs, etc.) are in place to serve this purpose. For example, the doping sample must be collected at the right time (i.e., at a time at which the doping substance is detectable within the sample). Also, there must be knowledge of which prohibited substances are potentially being used by the athlete subject to testing. Moreover, the anti-doping laboratory's sample diagnostics must be reliable in detecting those targeted substances.

Machine learning approaches and Artificial Intelligence (AI), enable to integrate diverse data sources, may support ADOs' existing approaches of doping risk assessment based on data such as competition results, biological markers, or demographic factors of athletes (26, 27). Recent studies have leveraged machine learning and other AI techniques in areas such as performance monitoring, biomarker analysis (27, 28), or sample fraud detection (29). These techniques were used, for example, to flag anomalous performances or suspicious biological profiles indicative of doping and demonstrated improved accuracy compared to traditional approaches (27, 29). However, the effectiveness of such AI-based methods depends heavily on the quality and relevance of the underlying data and should be complemented by domain-specific insights and established profiling methods (30). When applied with care, AI techniques and machine learning have the potential to support more nuanced doping risk profiling, improve the timeliness and accuracy of doping detection, and enhance the overall effectiveness of ADOs' testing strategies. Still, testing effectiveness in detecting doping depends on intelligence, knowledge, algorithms, and diagnostics available at the specific point in time the risk assessment of testing regimes is conducted (22).

To bridge deficits in intelligence, knowledge, algorithms, or diagnostics at that given point in time, WADA allows ADOs to store collected anti-doping samples for up to ten years for future re-analysis, referred to as sample retention and further analysis (SFA) (22, 31). ADOs may initiate the re-analysis of a stored sample, allowing them to retrospectively exploit newly gathered intelligence on doping or improved detection diagnostics and increase the long-term detection potential of testing (31). Literature evidence supports this approach. For example, analyzing data on Anti-Doping Rule Violations (ADRVs) in international weightlifting, Kolliari-Turner et al. (32) showed that 61 weightlifters competing in the 2008 and 2012 Olympic Games produced retrospective ADRVs due to the discovery of long-term metabolites in the targeted re-analysis of retained doping control samples, with 34 original medalists among the convicts. Kolliari-Turner et al. (33) extend the analysis to ADRVs that have impacted medal results at the Summer Olympic Games from 1968 to 2012, finding that 57% of all 134 ADRVs impacting medal results were uncovered in the course of the SFA application. These findings underline SFA's ability to enhance the long-term reliability of doping detection.

Recalling testing's second goal of reducing doping prevalence by imposing deterrence (17), deterrence theory postulates that a higher (1) certainty, (2) severity, and (3) celerity of punishment consequential to a crime leads to lower rates of criminal behavior (19). From an athlete's perspective, more reliable testing regimes increase the perceived certainty of being caught and punished for an anti-doping rule violation (ADRV), influencing an athlete's doping decision (18, 34). In sight of SFA application, the celerity (or speed) of uncovering an ADRV through testing and imposing sanctions subsequently becomes relevant as another dimension of effective deterrence due to allowing re-analysis of stored anti-doping samples for up to 10 years (22). Following deterrence theory, punishments or sanctions consequential to criminal acts should arrive sooner rather than later after the offence to increase deterrence (35). Consequently, SFA application presents a trade-off between certainty and celerity of sanctions. The longer anti-doping samples are stored, the higher the probability of diagnostics improvement or new intelligence coming to light that can be exploited for doping detection (as illustrated by Kolliari-Turner et al. (32), but the lower the celerity of sanctions consequential to an ADRV and the least it serves as an effective deterrent.

Underlining this complex trade-off, Westmattelmann et al. (34), surveying 146 elite athletes, highlight that SFA, despite its ability to effectively detect doping retrospectively (32, 33), is perceived by athletes as only a moderately effective anti-doping measure. Athletes emphasize that “lost moments” (such as medal ceremonies and media recognition following major victories) cannot be fully restored after several years, and any financial compensation provided is typically insufficient (27, 34, 36, 37). Similarly, Kuuranne and Saugy (38) stress the importance of the timeline defined for retesting in maximizing SFA's deterrent effect. Using a game-theoretic interaction model, Goetsch and Salzmann (39, 40) explore how the implementation of SFA influences athletes' doping intensity within the framework of an anti-doping testing strategy. They theoretically establish the existence of a “doping-minimizing retesting scheme” and propose that a coordinated, strategic application of SFA achieves greater deterrence than randomized testing.

The discrepancy between actual doping behavior (represented by the estimated doping prevalence rate) and detected doping (represented by the share of positive test results) necessitates further assessments of testing effectiveness. Previous research on the effectiveness of anti-doping measures has focused on athletes' perceptions [e.g., (34, 41, 42)], which do not reveal how the extent of implementation affects athletes' choices regarding doping use, and, ultimately, the prevalence of doping. Only a limited number of studies were conducted on athletes actually sanctioned for ADRVs, with mixed results. Kirby et al. (43) and Engelberg et al. (44) reveal that both individual psychological factors and broader cultural influences play significant roles in doping decisions, with athletes emphasizing that, e.g., guilt of shame were predominant deterrents to doping rather than testing and sanctions. Furthermore, these studies indicate that doping tends to begin early in athletes' careers and is normalized within certain sporting cultures, complicating the establishment of effective deterrents. Cox et al. (45) highlight similar issues among a sample of Welsh rugby players, where concerns about the effectiveness and legitimacy of doping controls underscore the need for more research into the effectiveness of testing regimes.

Quantifying the effectiveness of anti-doping measures like SFA and their effect on doping prevalence presents a major challenge since reliable estimates of doping prevalence are lacking (16), and no reliable indicator to measure anti-doping effectiveness exists (46). To overcome the significant challenge of measuring the effectiveness of long-term storage and sample retention—given the lack of reliable empirical data and indicators—this study employs an agent-based modelling approach. Agent-based modelling allows for the simulation of individual athletes' doping behaviors and their interactions within an artificial environment, capturing the complex dynamics of how athletes respond to different anti-doping measures over time (47). By modelling these micro-level decisions and interactions, agent-based modelling enables us to predict and quantify the impact of various SFA strategies on both doping prevalence and detection rates (48, 49). This approach not only helps to fill the gaps left by insufficient empirical estimations but also aids in identifying effective strategies for long-term storage and sample retention. In doing so, this study aims to quantify how SFA contributes to testing's dual objectives of deterrence (represented by SFA's influence on actual doping behavior) and detection (represented by SFA's influence on the share of detected doping). The following research question guides the investigation:

What effect does SFA application in anti-doping testing have on doping behavior and detected doping?

To address this research question, we employ an agent-based modelling approach to simulate the impact of SFA application on both doping behavior and detection rates among athletes. The study is structured to first detail the methodology behind the agent-based model, including the design of the model and the parameters used for simulation. We then present the results of our simulations, highlighting how variations in the number of retested samples and storage durations influence doping prevalence and detection. This is followed by a discussion of the findings in relation to existing literature, emphasizing the implications for both research and anti-doping practice. Finally, we acknowledge the limitations of our study and suggest avenues for future research to enhance the effectiveness of anti-doping measures further.

## Materials and methods

### Methodology

This study seeks to predict how athletes’ doping behavior changes in response to SFA strategies implemented by ADOs. To achieve this, we develop an Agent-Based Model (ABM). The ABM approach allows us to simulate a social system where interactions among members result in emergent behavioral patterns that cannot be fully understood by isolating individual behaviors (50). This makes agent-based modelling a suitable method for capturing the complexity of these interactions (51). Furthermore, ABM provides a dynamic framework that more closely mirrors real-world scenarios (52). Within this model, entities, referred to as agents, interact in the social system according to predefined rules, random elements, and diverse decision-making processes (53). The capacity of agents to factor in environmental conditions and the behavior of others when making decisions allows for a certain degree of autonomy in their actions (50). As athletes make decisions regarding doping by considering the behavior of other athletes, as well as potential rewards or penalties related to competition outcomes, agent-based modelling is a suitable tool for analyzing doping behavior in elite sports with a high level of complexity (49).

### Model description

The ABM integrates insights from previous research on anti-doping as well as established practices in the field. The primary objectives are twofold: first, to create a model that accurately mirrors real-world scenarios, and second, to identify behavioral tendencies or changes when varying model parameters related to SFA application. Our Doping ABM simulates the interactions between four key entities: (i) a sports event organizer, (ii) an anti-doping organization, (iii) an anti-doping laboratory, and (iv) athletes.

The **Sports Event Organizer** is responsible for planning and executing a specific sports event, setting the prize money (PM), and paying athletes based on their final rank in the competition (54).

The **Anti-Doping Organization** (ADO), which represents institutions like WADA, national anti-doping agencies, or (International) Sports Federations, oversees doping prevention in competitive sports. The ADO sets the Complexity of Anti-Doping Rules (CAR), determining how intricate the rules are for athletes to follow. Additionally, the ADO enforces sanctions, such as bans (BAN) for athletes caught using performance-enhancing drugs and may impose fines (FIN). Given that real-world ADOs, as well as federations at both national and international levels, have the authority to sanction athletes [see (1)], this assumption is well-founded. For simplicity, the model explicitly includes the role of ADOs in administering sanctions while implicitly acknowledging the overlapping authority of federations. Athlete agents, thus, face a system of punishment, which serves as a potential deterrent to doping.

The **Anti-Doping Laboratory** is tasked with conducting doping tests, much like WADA-accredited laboratories [see, (1)]. Two types of testing are considered: (1) regular testing, where all collected samples are analyzed, and (2) long-term storage and retesting of a subset of these analyzed samples. In regular testing, the top three athletes by performance are tested (55). In addition, while real-world testing is based on testing plans developed through risk assessments and includes athletes from the Registered Testing Pool (RTP) who are prioritized for testing more frequently (22), this model simplifies the selection process by randomly selecting additional athletes for testing, irrespective of their rank (56, 57). This approach was chosen for three reasons. First, publicly available information on the procedures that ADOs use for targeted testing and SFA allocation is extremely limited, so calibrating a realistic dynamic strategy would require unverifiable assumptions. Second, a constant selection procedure allows us to isolate the marginal deterrent effect of SFA without conflating results with complex, uncertain selection heuristics. Finally, a parsimonious representation keeps the model tractable and the findings interpretable, thereby providing a transparent baseline for subsequent extensions. This simplification aligns with the aim of modeling general trends rather than replicating exact operational processes. In this model, the number of athletes tested regularly (NTE; Number Tested) is fixed at 10. Thus, the top three athletes are always tested, with seven more selected randomly. Future versions of the ABM can replace the fixed NTE parameter with a risk-based sampling function that maps athlete- or sport-specific indicators (e.g., performance variations, whistleblower information, biological passport irregularities) to testing probabilities once those indicators are empirically validated. Moreover, while all collected samples must be analyzed immediately under current regulations, a portion of these samples analyzed may also be stored for long-term retesting, in accordance with WADA guidelines. The ADO determines how many of these samples will be stored (retesting_NTE) and the duration for which they will be stored (stored periods). As previous studies [e.g., (58)] indicate, anti-doping tests are not perfect since not all substances and methods can be detected at any time, and this is reflected in our model. Doping controls (both regular and retests) conducted by the Anti-Doping Laboratory are imperfect, meaning not all doped athletes are caught even if tested.

Our model assumes that diagnostic testing improves over time as analytical techniques advance. In line with Westmattelmann et al. (49), the control efficiency for regular testing is set at 20%. However, the control efficiency for retesting (retesting_CEF) increases incrementally, improving by 0.1 every two periods for stored and retested samples, so that after two periods, it is 0.2; after four periods, 0.3; and after eight periods, 0.4. In diagnostic-testing terminology, the control-efficiency parameters (CEF, retesting_CEF) capture the probability of a false negative, i.e., a doped athlete who is not identified even when sampled and tested. This may occur in instances where prohibited substances or methods and their associated markers cannot be identified through the utilised testing methodology. Alternatively, a false negative of a doped athlete may occur when the substance is actually not present in the sample, due to the collection of the sample occurring outside the detection window of the targeted doping substances or methods. The impact of false negatives on an athlete's doping decision can be attributed to the “rational” athlete behavioral type, explained in the following. A complementary event, the false-positive (clean athlete incorrectly declared doped), is not modelled because its empirical frequency in WADA-accredited laboratories is very low, and every presumptive adverse finding is confirmed (or refuted) through an obligatory B-sample analysis if requested by the tested athlete (59). Nevertheless, we acknowledge that even a very small false-positive risk may influence athletes' subjective perceptions of procedural justice. To make this explicit, we assume the probability of a false positive to be 0 in the baseline model.

The Athletes in the model are heterogeneous in their attributes, with their main objective being income generation through participation in competitions. The income is dependent on their ranking (RR) in the competitions (similar assumptions can be found in (57, 60, 61). An athlete's rank is a reflection of their performance (PF) relative to other competitors. Performance is determined by three factors: Fitness (FI), representing an athlete's talent, which can be enhanced through training; Constitution (CO), representing their overall physical condition; and a disturbance factor (DI), a random variable accounting for external factors during the competition. Each of these components is weighted (WF for fitness, WC for constitution, and WD for disturbance) in the model (49). Hence, the athlete's performance can be defined in Equation 1 as:

(1) $$\begin{array}{rl} & PF=\;WF*FI+WC*CO+WD*DI\\ & subject\;to:\;WF+WC+WD=1;\;WF,\;WC,\;WD\geq 0 \end{array}$$

At the start of the simulation, each athlete is randomly assigned values for FI, CO, and DI within a range of 0–100. In subsequent periods, FI and DI can be influenced by the athlete's decision to use doping, while DI remains randomly determined in each period. FI reflects an athlete's short-term condition, whereas CO represents long-term physical health. Doping harm (DH) is introduced in the model, as doping use is assumed to deteriorate the athlete's constitution over time. Eber and Thépot (62) introduce the concept of health costs in their model, and Birzniece (63) provides a comprehensive review of medical studies highlighting the long-term effects of doping. In this model, doping harm increases after doping use reaches a peak and then gradually diminishes in the following periods.

The model also incorporates doping efficiency (DE), as numerous studies suggest that doping enhances short-term fitness [e.g., (60, 61)]. In our simulation, doping efficiency spikes immediately after doping use and then gradually fades over the next two periods. To calculate an athlete's income for any given period (IP), the model considers their prize money (PM) alongside potential expenses like fines or doping costs, which are influenced by their decision to dope (DO). Based on Haugen et al. (64), the model distinguishes between three income scenarios:

1. The athlete dopes but is not caught. In this case, income equals prize money from doping (PM_+) minus doping costs (DC).
2. The athlete dopes and is caught. Here, income equals PM_+ minus the penalty (LO), such as fines or a ban, and DC.
3. The athlete does not dope, resulting in income solely from prize money (PM_-).

Lastly, following Hokamp and Pickhardt (65), who applied an ABM to tax evasion, athletes in this model fall into one of four behavioral types (BT): A-Type (rational), who weighs the costs and benefits of doping (2); B-Type (suggestible), whose doping decisions are influenced by their social environment; C-Type (compliant), who refrains from doping due to moral reasons; and D-Type (erratic), who may dope unintentionally due to a lack of knowledge about anti-doping rules or mistakes in handling potential contaminants that can lead to an AAF unintentionally. B-type athletes, in particular, form social networks that may include athletes from other behavioral categories.

### Model execution

**Figure 1:**
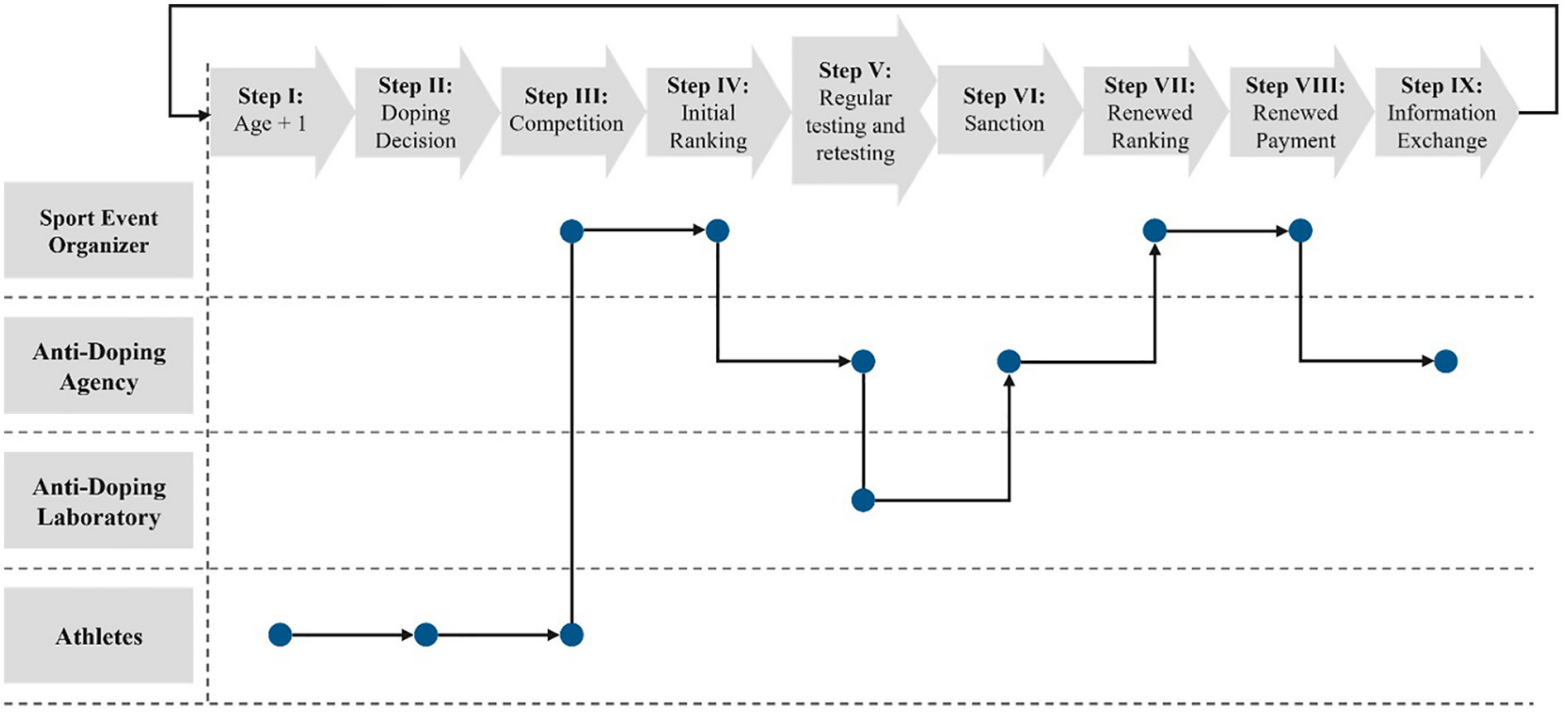
Simulation process in the agent-based model for doping behavior and detection. Source. In accordance with Westmattelmann et al. (49).

The model operates through a nine-step process in which the four entities described earlier interact, adhering to predefined rules and characteristics associated with their behavioral type. These characteristics are set at the beginning of the simulation process. The first step in the simulation process is to increase the agents' age by 1 year. Athletes who retire due to age are replaced by younger athletes who enter the system with the same initial attributes. Before the competition begins, athletes decide whether to engage in doping for the upcoming competition. This decision is influenced by their specific behavioral type, individual circumstances, and experiences from previous simulation rounds. After this decision-making phase, the competition takes place, and an initial ranking is generated based on the athletes' performance. Once the competition concludes, the ADO implements its testing plan, which consists of two parts. First, athletes are selected for regular testing based on both the competition results and a randomized testing strategy. In the second part, the ADO decides how many of the samples collected during regular testing will be placed in long-term storage and for how many storage periods these samples will be kept. Following this, the analysis of samples from the current period is executed by the Anti-Doping Laboratory, along with the analysis of stored samples that are scheduled for retesting after their designated storage duration has expired. Athletes caught using banned substances or methods based on regular or retesting are sanctioned by the ADO. These sanctions result in disqualification, which leads to a revised ranking of the current competition if doping was detected based on regular testing or to a revised ranking of a competition finished a couple of periods ago based on retesting. Based on these updated rankings, the prize money is redistributed among the athletes who were not disqualified. Following this, the ADO publishes statistics related to doping cases. The process then repeats for subsequent periods. For a more detailed description of each simulation step, please refer to Westmattelmann et al. (49). Figure 1 illustrates the overall simulation process.

The Agent-Based Model (ABM) was developed using NetLogo, version 6.1.1 (66). In the simulation, the number of stored and retested samples (Retesting_NTE) was varied across five levels [2, 4, 6, 8, 10, while the storage duration (Stored periods) was varied across four levels [2, 4, 6, 8]. These different combinations of stored and retested samples, along with their storage durations, were simulated in a total of 20,000 runs. Each simulation produced two key outcomes:

1. Actual doping behavior is represented by the prevalence rate, i.e., the share of athletes in the population who engaged in doping.
2. Detected doping reflects the share of athletes who were caught using banned substances during testing.

### Model calibration

To ensure that our model accurately reflects real-world conditions, we follow the parameter set proposed by Westmattelmann et al. (49), who calibrated their ABM with empirical evidence wherever such data were available. Accordingly, for example, BANned periods (BAN) were set to 4 periods, as according to the WADC, a ban of up to four years is imposed for first-time doping offenses (1). Following this, we assessed the effects of different retesting strategies by varying the number of stored and retested samples (retesting_NTE) and the storage periods to evaluate their influence on both the share of doped athletes (prevalence rate) and the proportion of detected athletes.

We began by simulating a status quo scenario to verify whether the model could replicate a realistic setting. In this baseline scenario, all four measures were set at 100% effectiveness. Specifically, this scenario included an NTE of 10 athletes, a CEF of 20%, FIN set to 100 tokens, a BAN lasting four periods, a retesting_NTE of 4, and a storage duration of four periods. To ensure comparability across all simulated scenarios, the distribution of behavior types was kept constant: A-type athletes made up 40%, B-type athletes 30%, C-type athletes 20%, and D-type athletes 10% of the sample. For a detailed overview of the parameter settings used in the model calibration, refer to the Appendix Table A1.

### Empirical plausibility checks

Research on doping prevalence highlights a wide range of reported doping rates in competitive sports. These large discrepancies are driven by high variability in athlete populations, with factors like gender, sport-specific demands, or regional disparities in anti-doping enforcement playing a key role. Moreover, methodological variation across studies on doping prevalence distorts comparability and the derivation of a robust estimation (16). Prevalence estimates vary between 0% and 66.7% based on self-reports and 0%–48% based on biological markers, highlighting challenges of deriving a robust and meaningful empirical estimation of doping prevalence. Within the ABM, the status quo scenario simulation output yielded a share of doped athletes of 26.15%. While our model does not aim to predict the true doping prevalence precisely, the prevalence rate yielded by the status quo simulations underlines that the ABM provides an empirically sound framework that allows us to observe general trends in how SFA application influences doping behavior.

**Figure 2:**
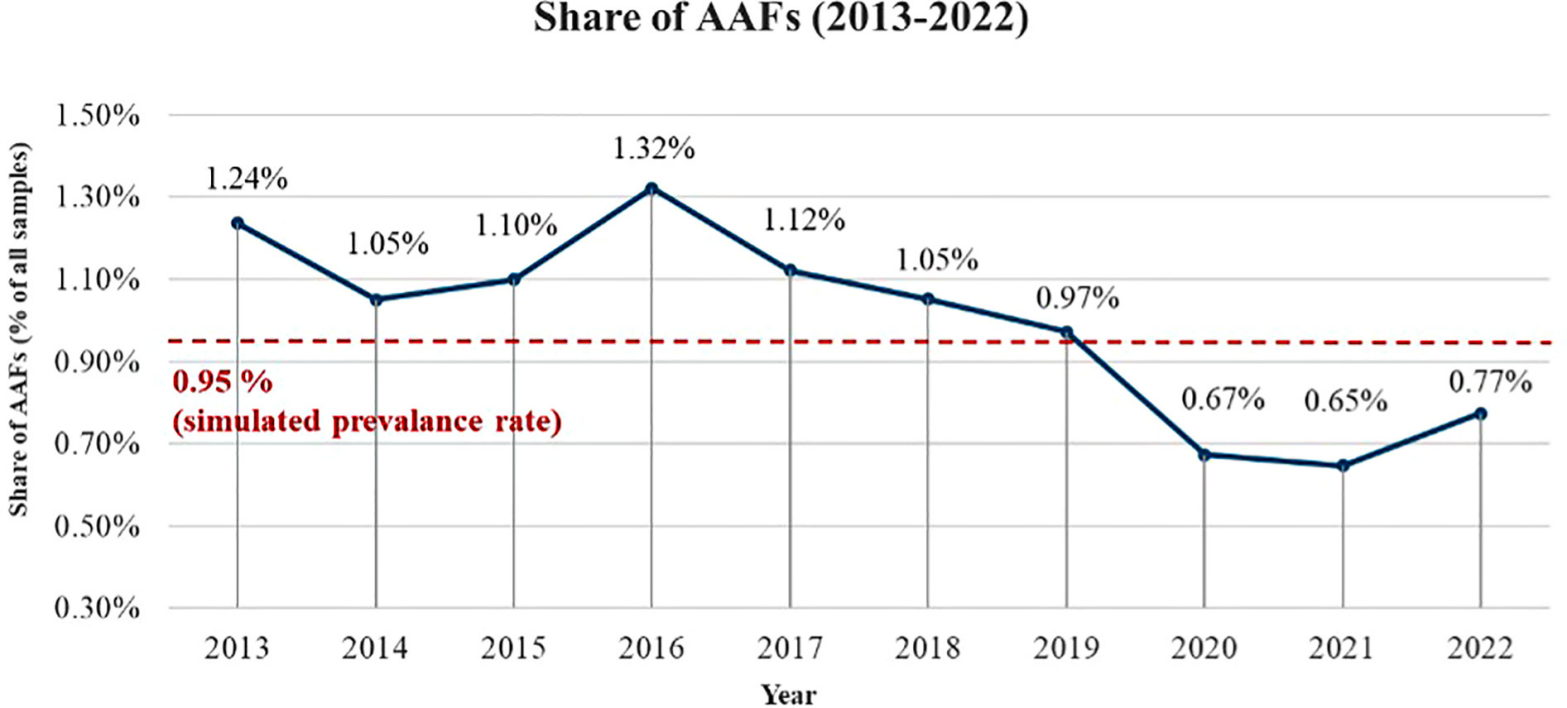
Share of AAFs (2013–2022). Source. In accordance with WADA (6–15) results.

Regarding the detection of doping, the share of detected athletes in the status quo scenario simulation was on average 0.95%. This detection rate, as the second output of the simulation model, aligns closely with the actual shares of Adverse Analytical Findings (AAF) fluctuating around 1% reported by WADA since 2013 and illustrated in Figure 2. Thus, the share of detected athletes of 0.95% in the model can be considered a reliable representation of real-world anti-doping enforcement, also allows us to observe general trends in how SFA application influences doping detection. Note that AAFs depicted in Figure 2 are inclusive of TUEs, which accounts approximately 10% of all athletes with AAF, and athletes can have more than one test, and more than one AAF, especially with TUE, in any given year.

The analysis of the simulation results consists of two parts. First, the influence of the number of stored and retested samples, the storage duration, and a simultaneous variation of both measures on the prevalence rate and the proportion of detected doping is visualized using two sensitivity analyses. Second, the effect of stored and retested samples and storage duration on both the prevalence rate and the proportion of detected doping is quantified using regression analysis.

### Sensitivity analysis

**Figure 3:**
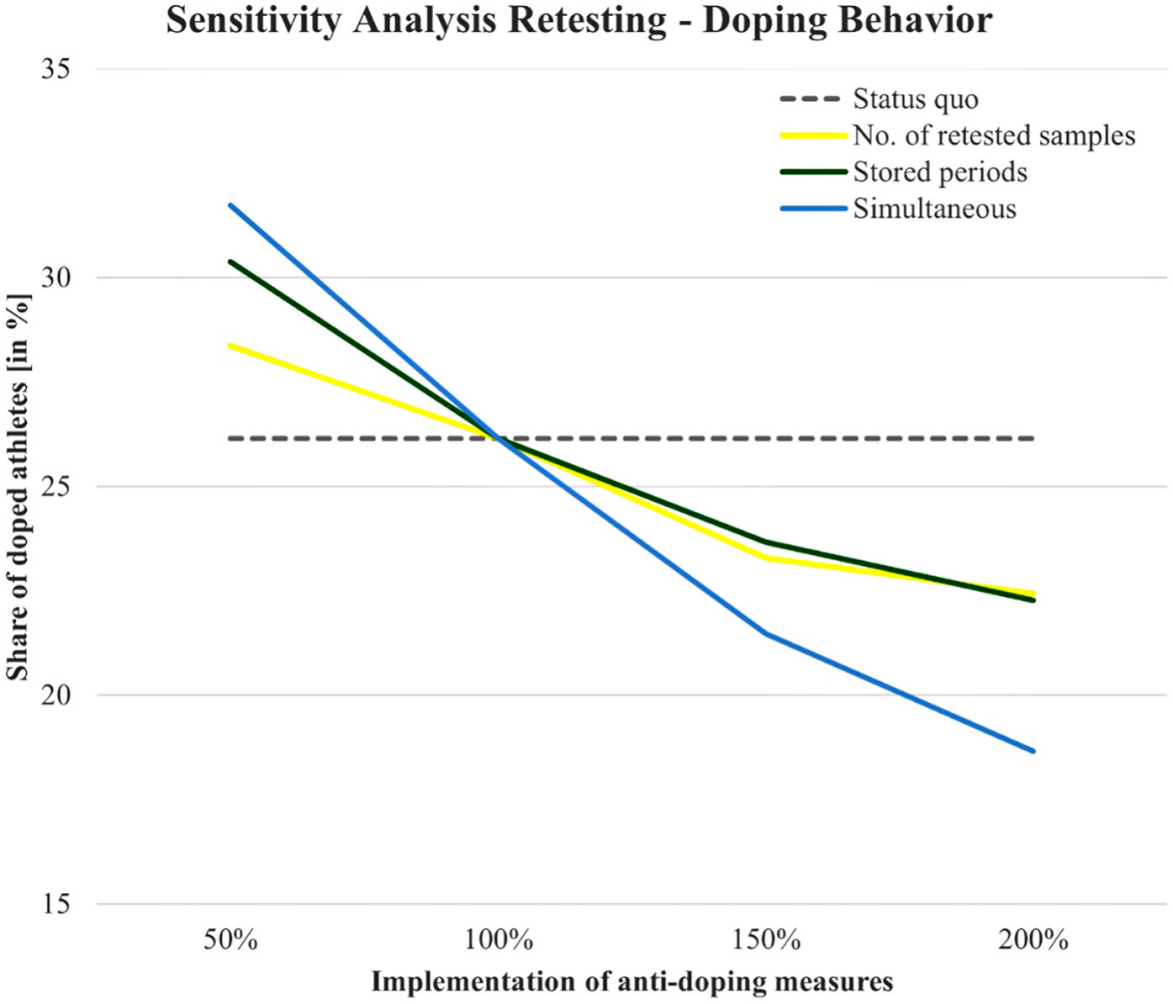
Impact of retesting strategies on doping behavior: sensitivity analysis results.

The sensitivity analysis conducted in this study aims to explore the influence of two critical factors—(1) the number of stored and retested samples and (2) the storage duration—on both doping behavior and the detection of doping. This analysis examines how varying these two measures impacts the share of doped athletes and the share of detected doping in the population over time. Figures 2, 3 illustrate the outcomes of these variations using three scenarios: number of retested samples, stored periods, and simultaneous variation of both.

### Doping behavior

As shown in Figure 3, the sensitivity analysis reveals a clear inverse relationship between the implementation of anti-doping measures and the share of doped athletes. Across all scenarios, as the number of retested samples and storage periods increases, the prevalence of doping behavior decreases. Notably, the simultaneous variation of both retesting and storage periods (blue line) shows the strongest effect in reducing doping behavior, suggesting that a combined approach is most effective. In contrast, the effect of adjusting the number of stored and retested samples alone (yellow line) or storage periods alone (green line) is less pronounced but still discernible. Increasing either measure individually results in a gradual decline in the share of doped athletes. Here, the impact of increasing storage periods is slightly larger than the impact of increasing the number of stored and retested samples, illustrated by the slightly steeper overall gradient of the green line.

### Detected doping

**Figure 4:**
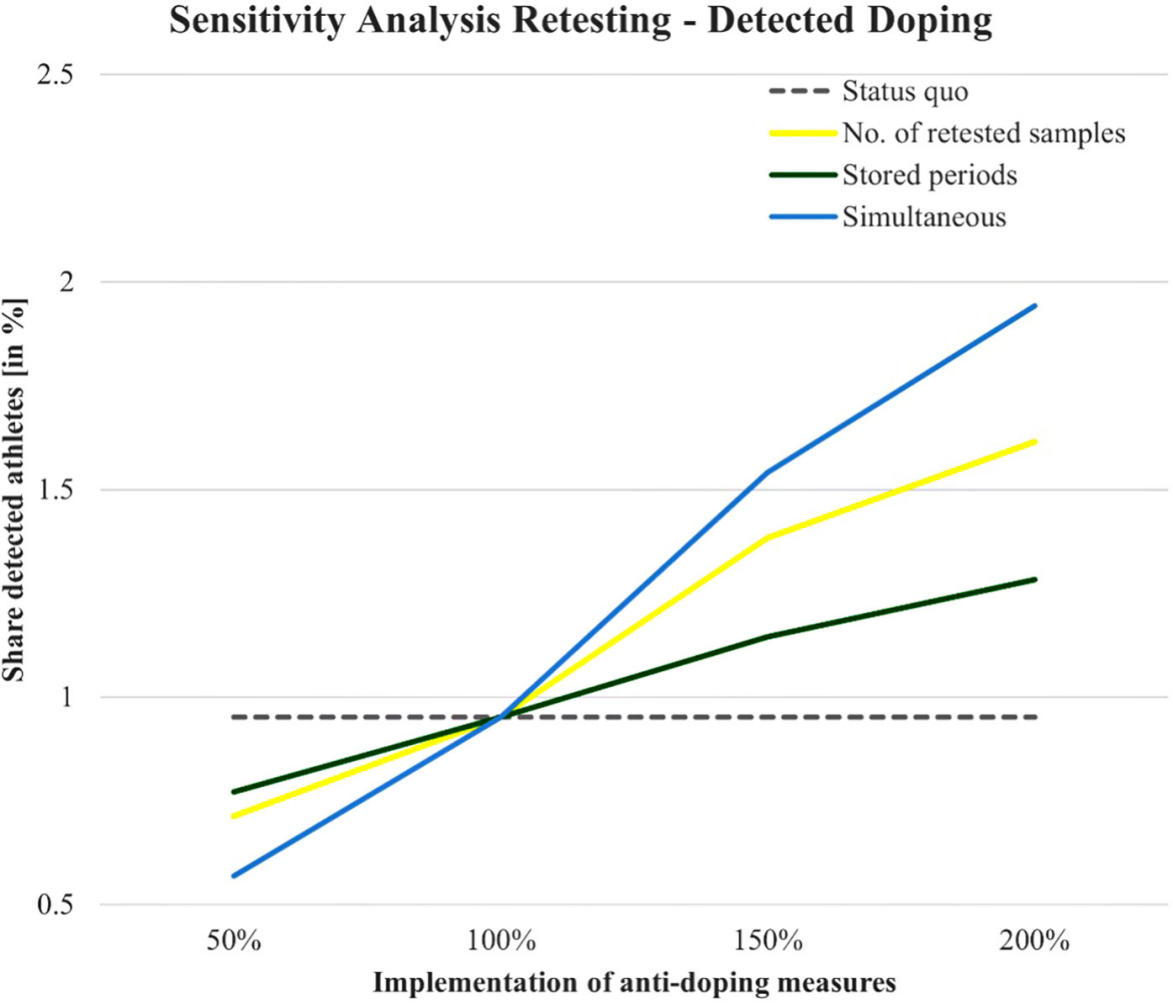
Impact of retesting strategies on detected doping: sensitivity analysis results.

The effect of retesting strategies on detected doping is illustrated in Figure 4. Here, we observe a positive effect of the intensity of anti-doping measures on the share of detected doping cases. Like the doping behavior results, the simultaneous variation scenario (blue line) produces the most significant increase in detection rates, reaching over 2% at the highest levels of implementation. This indicates that combining both the number of retested samples and storage duration significantly enhances the detection of doping over time. The number of retested samples alone (yellow line) also has a considerable effect on detected doping, with detection rates increasing steadily as more samples are stored and retested. On the other hand, varying the storage periods alone (green line) produces a more gradual increase in detection rates, suggesting that the number of retested samples is a more critical factor than storage duration when it comes to improving detection.

Overall, the sensitivity analysis demonstrates that both the number of stored and retested samples and the storage duration have a significant impact on doping behavior and detection. However, a combined approach that simultaneously increases both measures yields the most effective results, reducing the prevalence of doping while substantially increasing the detection rate.

### Quantifying effectiveness

To quantify the effect of the number of retested samples (Retesting_NTE) and storage duration (StoredPeriods) on both the share of doped athletes and the share of detected athletes, we conducted two separate regression analyses. These analyses use transformed predictor variables by taking the square root of Retesting_NTE and StoredPeriods, which accounts for the non-linear relationship observed in the sensitivity analysis. This transformation helps to capture the diminishing returns of increasing the number of retested samples and storage durations on doping prevalence and detection rates. As the sensitivity analysis demonstrated, the relationship between these anti-doping measures and the share of doped and detected athletes is not perfectly linear. Initially, small increases in retested samples or storage duration lead to significant changes in doping behavior and detection rates, but the marginal effects decrease as these measures are scaled up. This finding is reflected in the non-linear regression model, where the square root transformation accounts for the non-linearity.

$$\mathrm{Share}\;\mathrm{of}\;\;\mathrm{doped}\;\mathrm{athletes}=a+b*(\mathrm{retesting}\mathrm{\_NTE}{)}^{\wedge }0,5+c*(\mathrm{StoredPeriods}{)}^{\wedge }0,5+\varepsilon$$

$$\mathrm{Share}\;\mathrm{of}\;\;\mathrm{detected}\;\mathrm{athletes}=a+b*(\mathrm{retesting}\mathrm{\_NTE}{)}^{\wedge }0,5+c*(\mathrm{StoredPeriods}{)}^{\wedge }0,5+\varepsilon$$

These equations indicate that the dependent variables (share of doped athletes and share of detected athletes) are modelled as a function of the square root of the independent variables Retesting_NTE and StoredPeriods, plus a constant (a) and an error term (*ε*). The regression coefficients (b and c) represent the rate of change in the share of doped or detected athletes with respect to each predictor, but at a diminishing rate, consistent with the non-linear trends observed in the sensitivity analysis. This approach allows for a more accurate representation of how these anti-doping measures influence doping behavior and detection over time.

### Share of doped athletes

The first regression analysis examined the relationship between the number of retested samples, storage duration, and the share of doped athletes in the population. As shown in Table 1, both predictor variables, the square root of Retesting_NTE and the square root of StoredPeriods, had significant negative effects on the share of doped athletes. The model's constant was 44.78, indicating that, without any retesting or storage interventions, the baseline doping prevalence would be around 44.78%.

**Table 1:** Regression results share of doped athletes.

Predictor	B	SE	T	P	95% CI
Constant	44.78	0.19	233.9	<.001	[44.40, 45.17]
sqrt(Retesting_NTE)	−0.38	0.05	−7.76	<.001	[−0.47, −0.29]
sqrt(StoredPeriods)	−0.62	0.06	−11.28	<.001	[−0.73, −0.52]

The coefficient for Retesting_NTE was −0.38 (*p* < .001), meaning that as the number of retested samples increased, the share of doped athletes decreased. Similarly, the coefficient for StoredPeriods was −0.62 (*p* < .001), demonstrating that longer storage durations also led to a reduction in doping prevalence. Both factors contributed to a statistically significant model, with an *R*^2^ value of 0.095, indicating that approximately 9.5% of the variance in doping behavior could be explained by the retesting and storage strategies [F(2, 19,998) = 1,054.2, *p* < .001].

### Share of detected athletes

In the second regression analysis, the same predictor variables were used to analyze their effect on the share of detected athletes. As reported in Table 2, both variables showed a significant positive effect on detection rates. The constant for the model was −1.09, suggesting that without retesting or storage, the detection rate would be close to zero, as expected, given the limitations of immediate testing.

**Table 2:** Regression results share of detected athletes.

Predictor	B	SE	t	P	95% CI
Constant	−1.09	0.06	−19.93	<.001	[−1.19, −0.98]
sqrt(Retesting_NTE)	0.66	0.02	42.79	<.001	[0.63, 0.69]
sqrt(StoredPeriods)	0.41	0.02	22.63	<.001	[0.37, 0.44]

The coefficient for Retesting_NTE was 0.66 (*p* < .001), indicating that increasing the number of retested samples significantly raised the share of detected athletes. Likewise, the coefficient for StoredPeriods was 0.41 (*p* < .001), meaning that longer storage periods also contributed to higher detection rates. The model explained 10.5% of the variance in detection outcomes, with an *R*^2^ of 0.105 [F(2, 19,998) = 1,172.0, *p* < .001].

## Discussion

Using an ABM, we quantified how variations in storage duration and the number of retested samples influence athlete doping behavior and detection efficacy. The sensitivity analysis and regression models revealed that the number of stored and retested samples, along with the duration of storage, significantly influence doping behavior and detection outcomes in the implemented ABM. How ADOs could improve the effectiveness of their testing regimes requires the right combination of sample storage and retesting policies, tailored to the specific context (e.g., country and sport-specific parameters). Various scenarios to facilitate the contextually best strategies for SFA application are discussed below.

### Effectiveness of varying storage duration and number of retested samples

When examining the effects of increasing the storage duration and number of retested samples in isolation, an inverse relationship emerges: An increase in storage duration has a greater impact on reducing doping behavior. In contrast, an increase in the number of retested samples has a stronger effect on increasing the detection rate. This relationship is also reflected in the regression analysis, where storage duration proves comparatively more effective in reducing doping behavior, whereas the number of retested samples is more effective in enhancing the detection rate. ADOs can leverage this insight to design their testing regimes strategically.

Following deterrence theory, the effect of increasing storage duration on doping behavior implies that athletes perceive the increasing certainty of detection over time (e.g., through novel testing methods or advancements in intelligence and investigations) as a particularly effective deterrent (19). Thereby, the deterrent effect of increased certainty of sanctions outweighs the negative impact of decreased celerity (swiftness) of sanctions. Increasing storage duration consistently exerts a negative effect on doping behavior. However, sensitivity analysis shows that the effectiveness of longer storage durations diminishes over time: The higher the storage duration, the flatter the marginal effects on doping behavior if storage duration further increases. This can be rationalized from the athlete's perspective: If no sanctions are feared over several years due to prolonged storage durations, the short- to medium-term advantages of doping become more attractive. In professional sports, short- to medium-term career planning is predominant because of 4-year Olympic cycles (67) or even yearly major championships [e.g., in athletics, (68)]. Larger prize money can be realized more immediately and with greater certainty due to short-term doping advantages, while the discounted value of a sanction decreases as its occurrence recedes further into the future. This is especially relevant for athletes at the end of their career, as the perception of risks, e.g., regarding the consequences of prolonged bans from competition, is lower compared to athletes at the beginning of their career (49). Nonetheless, the marginal effect of extending storage duration remains positive, even at already high levels.

Higher numbers of stored and retested samples are effective in increasing the detection rates of doping. As underlined by Kolliari-Turner et al. (33), a majority of ADRVs impacting Olympic medal results can be attributed to the re-analysis of stored samples. This can be explained based on the suboptimal efficiency of doping controls, concerning available knowledge on doping, as well as limited intelligence insights. Storing more samples for future re-analysis using improved detection methods and leveraging gathered intelligence likely increases the chance of positive findings through more sophisticated, targeted retesting. The simulation results indicate that increasing the number of stored and retested samples can be an effective long-term strategy to enhance detection rates.

The results suggest that a combined approach, simultaneously increasing both storage duration and retesting frequency, yields the most effective outcomes in reducing doping behavior and increasing the detection rate over time. ADOs employing SFA should recognize that an intelligent combination of extended storage durations and strategic retesting is the most effective approach to reduce doping behavior and enhance doping detection simultaneously. These findings provide implications for developing effective testing strategies considering SFA implementation.

## Contributions and implications

### Methodological

The employed ABM presents a novel and robust methodological approach in sports management research to quantify the effectiveness of anti-doping measures, specifically SFA application in testing regimes. This provides a substantial methodological contribution to research aiming to quantify anti-doping effectiveness, relating implementation levels of anti-doping measures to two outcome variables: doping detection rate and the doping behavior of athletes. By establishing doping behavior as a quantitative indicator of testing's ability to deter doping practices, the research addresses previous calls for clear indicators to measure doping deterrence [e.g., (4, 17)].

Beyond its application to anti-doping, the ABM offers a flexible framework that can be adapted to study other areas where individual decisions impact collective outcomes. Importantly, the model allows for capturing complexity and interactions by enabling the simultaneous manipulation of multiple parameters, such as the number of stored and retested samples and the storage duration and observing their combined and non-linear effects. This approach reveals that the impact of one measure cannot be fully understood in isolation from the other, underscoring the importance of considering the entire system when evaluating anti-doping strategies.

Moreover, the ABM approach facilitates identifying optimal parameter combinations. The results show that improving both storage duration and the number of retested samples simultaneously leads to more pronounced effects than varying these parameters independently. This finding provides a methodological advancement in guiding decision-makers in anti-doping policy design, allowing them to strategically calibrate multiple measures rather than relying solely on incremental changes to individual parameters.

Even though comprehensive empirical validation against longitudinal data that simultaneously track athlete doping behaviour and detection trends under systematic SFA implementation is not yet possible, the model is built on assumptions that reflect the core architecture and processes of the anti-doping system—explicitly representing Sport Event Organizer, Anti-Doping Organization, Anti-Doping Laboratories, and athletes. Its calibration draws on the latest anti-doping governance instruments (e.g., the 2021 WADA Code, ISTI) and current empirical research, ensuring that behavioural and operational parameters are evidence-based (69). Consequently, the two model outputs—overall doping prevalence and detection rate—fall within empirically observed ranges reported for elite sport (7–16), underscoring their realism. Empirically testing whether the magnitudes of the SFA effects revealed by our simulations mirror real-world outcomes is, however, impossible with the data presently available. No longitudinal dataset links specific sample-retention parameters to subsequent ADRVs. Although case-based evidence such as Kolliari-Turner et al. (32, 33) indicates that SFA can uncover additional ADRVs, these fragmented data are insufficient for a formal validation of our system-level findings. This paucity of comprehensive field data highlights the necessity of alternative approaches like ABM, which allow researchers and policy-makers to explore and optimise anti-doping strategies that cannot yet be examined empirically.

### Theoretical

In quantifying the effectiveness of SFA application in reducing doping behavior and increasing detection rates, this study contributes to delineating the dual objectives of testing regimes (17). It provides actionable advice on how to adjust testing efforts targeted towards effective deterrence or reliable doping detection. From the perspective of deterrence theory (19), an increase in the certainty of punishment through SFA application, enabled by retaining doping control samples for future analysis, comes at the expense of the celerity of punishment. The simulation results suggest that increasing storage duration significantly contributes to the deterrence effect of SFA, suggesting that increased certainty of being caught doping and sanctioned outweighs the reduced celerity of sanctions.

Moreover, the findings highlight non-linear deterrence dynamics, demonstrating that the effects of SFA measures do not scale proportionally with their intensity. As implementation levels of SFA rise, the marginal gains in deterrence begin to decrease. This non-linear pattern enhances our theoretical understanding by indicating that simply increasing SFA measures without considering their diminishing returns may be less effective in the long run. Further, the results underscore the temporal dimension of deterrence, showing that the prospect of eventual detection—due to sample storage and the later application of improved diagnostics—shapes athletes’ decision-making beyond immediate competitive cycles. Over time, this introduces a forward-looking consideration for athletes, who must weigh the risk of being caught in the future against the short-term benefits of doping.

Thereby, a contribution to further understanding the dimensions of deterrence theory is given in the context of detection-based deterrence through anti-doping testing. Accordingly, the SFA application in anti-doping presents insightful evidence of celerity as a highly relevant dimension of deterrence outside the criminal justice system, as suggested by Pratt and Turanovic (70). This study advances the social science literature on anti-doping by quantifying the effectiveness of applying SFA in testing regimes. It directly aligns with the anti-doping research priorities highlighted in a Delphi study by Boardley et al. (20), which stressed the need to assess the effectiveness of anti-doping interventions and education programs.

### Practical

ADOs and (International) Sports Federations responsible for planning testing regimes should recognize that at the time of sample collection and initial testing, not all factors critical to effective doping detection are known. For instance, knowledge of abused substances is limited, and detection methods of anti-doping laboratories or (AI) algorithms supporting ADOs' doping risk assessment are still under development. Although intelligent and fast detection of doping through testing would be desirable, current testing statistics on low detection rates suggest that a satisfactory detection rate cannot be achieved in current testing regimes. Therefore, our results underline that designing testing strategies with a priority on SFA application can enhance the long-term effectiveness of detection.

If detection is focused, emphasis should be placed on extensive and targeted retesting of stored samples. Particular attention should be paid to the quality of collected doping control samples (e.g., simultaneous long-term storage of both urine and whole blood samples), which are robust in results management and well-established to be subject to a multitude of analytical approaches (58). Storage duration should be sufficiently long to enable effective detection. ADOs must carefully consider the right timing of targeted retesting, considering factors such as emerging intelligence or new detection methods of anti-doping laboratories that present promising opportunities for identifying doping violations. While advancements in artificial intelligence (AI) and machine learning offer promising tools for refining doping risk profiling, their use must be guided by specific intelligence and supported by meaningful data. As highlighted in recent studies [e.g., (30)], performance profiling based on physiological and competitive data may currently offer a more robust and validated approach. ADOs should therefore carefully consider not only emerging technologies but also the contextual relevance, interpretability, and quality of the data they are based on when determining the timing and targets for sample storage and retesting.

Conversely, if the aim is to reduce doping behavior through deterrence in the first place, the results suggest that samples should be stored for longer periods to maximize the deterrent effect. To achieve a sufficient deterrent effect within the overall athlete population, samples of a multitude, if not all, athletes of a testing pool should be stored for extended durations, making adequate storage capacity a priority. The number of samples retested should be sufficient to uphold the credibility of testing and sanctions as a deterrence mechanism. Analytical detection methods employed should be chosen appropriately, particularly when no specific evidence on doping exists for the retested samples and re-analysis is conducted for preventive reasons. For deterrence purposes, sample types that can be collected in large quantities and are easy to store for long periods, such as dried blood spot samples, are particularly suitable (71, 72).

Our results show that both the number of stored samples and the number of retested samples decrease the doping behavior and increase the detection rate. Those effects are strongest when SFA is (initially) introduced in a small scale and weaken with increasing scopes of implementation. In practical anti-doping work, ADOs must consider their unique conditions under which SFA shall be implemented (e.g., testing budget and personnel, laboratory and storage capacities, or number of athletes overseen in testing pools). As a general baseline, WADA provides a template for developing an SFA strategy compliant with current regulations (73). Especially for ADOs not currently implementing SFA, our results underline that a small-scale implementation with manageable complexity can be an effective anti-doping measure. Also, anti-doping laboratories or testing service providers offer flat fees for sample analysis, storage and retesting, facilitating their consideration in the ADOs anti-doping budget (3, 74). With larger-scale implementation and diminishing returns of SFA implementation, prioritization frameworks suitable to the ADOs unique conditions are essential. More sophisticated decision-support tools and (AI-assisted) algorithms are likely necessary to transfer SFA strategies compliant with current anti-doping regulations into actionable and effective test distribution plans under consideration of an ADOs unique budgets, costs, and resources. Additionally, effective communication of testing activities to athletes is crucial to make SFA a credible deterrent. Informing athletes about, e.g., sample storage durations, retesting procedures, and advancements in detection methods reinforces the perception of SFA as an effective measure against doping.

## Limitations and future research

The current simulation model primarily emphasizes testing and SFA application as detection-based deterrence measures, omitting other anti-doping measures, such as whistleblowing systems or anti-doping education to detect doping or prevent doping in the first place (34). Future iterations of the ABM could integrate these additional measures to offer deeper insights into their effectiveness. Nonetheless, any extensions to the model should be added incrementally to allow for a clearer understanding of emergent effects. Additionally, while the model's approach to selecting athletes for testing simplifies the real-world process by relying on random selection (beyond the top three performers), actual anti-doping testing plans are based on risk assessments and prioritize Registered Testing Pool athletes for more frequent testing. This simplification does not fully capture the risk-based, intelligent testing strategies employed by ADOs, where testing plans are continuously adapted based on, for example, intelligence, athlete performance profiles, or sport-specific doping risks. While our simplification facilitates model tractability, it limits external validity, and future iterations of the ABM could integrate intelligent testing strategies better mirroring real-world practices of ADOs. WADA testing statistics highlight that not all adverse analytical findings through testing are actual ADRVs (11). More specifically, although the model accounts for imperfect detection efficiency in the form of false negatives, we do not endogenize false-positive findings. While this reflects their very low empirical incidence, future ABM extensions could test how even minimal false-positive risks alter athletes' cost–benefit calculus.

Moreover, the ABM represents athletes through four behavioral archetypes (rational, suggestible, compliant, and erratic). Although these categories are grounded in empirical observations [see (49, 69)], they cannot encompass the full range of moral reasoning, psychological traits, and social influences that shape doping behavior. In reality, athletes may display mixtures of the four archetypes whose relative weight shifts across contexts and over time (49). Therefore, the current model does not capture dynamic moral disengagement processes or affective states that recent literature identifies as critical determinants of doping decisions (75, 76). Future versions of the ABM could move toward hybrid or continuous behavioral profiles and allow these to evolve longitudinally in response to life events or policy changes.

Finally, even though our model's simulated detection rate closely matches WADA's official statistics, and the estimated doping prevalence, while inherently difficult to measure, is reasonably accurate (16), simulation models for prediction often face criticism [e.g., (77)]. The simulation results should, therefore, not be treated as empirical evidence but rather as quantitative indications of SFA effectiveness that may guide its practical implementation in ADOs testing regimes.

## Conclusion

This study underscores the potential of SFA implemented into anti-doping testing regimes, demonstrating its ability to inform anti-doping strategies that can both reduce doping prevalence and enhance detection rates. The findings highlight that longer storage durations effectively deter doping by increasing the perceived certainty of detection, while higher retesting frequencies are able to increase detection rates. Importantly, a combined approach yields the most impactful outcomes, reducing doping prevalence and increasing detection rates simultaneously. As the ABM simulation results are validated against real-world statistics, this study enables the derivation of actionable recommendations for ADOs on how to apply SFA in a variety of testing scenarios. Future research should explore the application of SFA together with complementary measures to provide a more comprehensive approach to doping deterrence. These insights contribute to advancing both research on the effectiveness of anti-doping work (17) as well as anti-doping practice.

## Data Availability

The raw data supporting the conclusions of this article will be made available by the authors, upon reasonable request.

## Appendix

**Table A1:** Overview of parameter setting for ABM.

Variable	Function	Value	Calibration
Sports event organizer			
PM	Prize money for tournament	[0; ∞]	100,000
Anti-doping organization			
CAR	Complexity of anti-doping rules	[0; 1]	0.2
BAN	Number of banned periods after being detected	[0; 8]	4
FIN	Fine after being detected	[0; 400]	100
Anti-doping laboratory			
CEF	Control efficiency	[0; 1]	0.2
NTE	Number of tested athletes	[0; N]	10
Retesting_CEF	Control efficiency of retesting	[0; NTE]	See analysis
Storage duration	Periods of sample storage	[0; 8]	See analysis
NDO	Number of doped athletes	[0; N]	Endogenous
SDO	Share of doped athletes	[0; 1]	Endogenous
NDE	Number of detected athletes	[0; N]	Endogenous
SDE	Share of detected athletes	[0; 1]	Endogenous
**Athletes**			
AD	Indicates whether athlete is detected	[“yes”;’ no’]	Endogenous
AG	Athlete's age	[minage, maxage]	At random
BA	Indicates whether athlete is banned	[0;1]	Endogenous
BT	Behavioral type	[A; B; C; D]	A = 0.4
			B = 0.3
			C = 0.2
			D = 0.1
CO	Athlete's constitution	[0; 100]	At random
DB	Decision value for comparison with CAR for BT D	[0; 1]	At random
DC	Doping costs	[0; ∞]	10
DE	Doping efficiency	[0; 1]	1
DH	Doping harm	[0; 1]	0.5
DI	Athlete's disturbance	[0; 100]	At random
DO	Doping decision	[‘-’; ‘+’]	Endogenous
FI	Athlete's fitness	[0; 100]	At random
FT	Athlete's initial fitness	[0; 100]	At random
ID	Unique ID for every athlete	[0; N-1]	Endogenous
IP	Income for respective period	[0; PM]	Endogenous
IW	Hypothetical income WITH doping	[0; PM]	Endogenous
IX	Hypothetical income WITHOUT doping	[0; PM]	Endogenous
LO	Loss by getting detected as doper	[0; PM]	Endogenous
PF	Athlete's performance	[0; maxperformance]	Endogenous
RD	Hypothetical rank if athlete is doped	[1; 100]	Endogenous
RN	Hypothetical rank if athlete is not doped	[1; 100]	Endogenous
RR	Realized rank	[1; N]	Endogenous
SD	Share of doping in network	[0; 1]	Endogenous
SN	Social network	[(list)]	At random
SO	Successful doping in social network	[0; 1]	Endogenous
SP	Subjective detection probability	[0; 1]	At random
SR	Subjective risk perception	[0; 1]	At random
SS	Size of social network	[0; N]	At random
TY	Tournament year	[1; 21]	Endogenous
UD	Utility for doping	[0; 1]	Endogenous
UN	Utility for NO doping	[0; 1]	Endogenous
WC	Weighting of constitution	[1—(WD + WF)]	0.4
WD	Weighting of disturbance	[1—(WC + WF)]	0.2
WF	Weighting of fitness	[1—(WC + WD)]	0.4

Source. In accordance with Westmattelmann et al. (49).
